# Evolutionary Rescue as a Mechanism Allowing a Clonal Grass to Adapt to Novel Climates

**DOI:** 10.3389/fpls.2021.659479

**Published:** 2021-05-17

**Authors:** Zuzana Münzbergová, Vigdis Vandvik, Věroslava Hadincová

**Affiliations:** ^1^Institute of Botany, Czech Academy of Sciences, Prague, Czechia; ^2^Department of Botany, Faculty of Science, Charles University, Prague, Czechia; ^3^Department of Biological Sciences and Bjerknes Centre for Climate Research, University of Bergen, Bergen, Norway

**Keywords:** alpine ecosystems, rapid evolution, reciprocal transplant experiment, trait selection, clonal species

## Abstract

Filing gaps in our understanding of species' abilities to adapt to novel climates is a key challenge for predicting future range shifts and biodiversity loss. Key knowledge gaps are related to the potential for evolutionary rescue in response to climate, especially in long-lived clonally reproducing species. We illustrate a novel approach to assess the potential for evolutionary rescue using a combination of reciprocal transplant experiment in the field to assess performance under a changing climate and independent growth chamber assays to assess growth- and physiology-related plant trait maxima and plasticities of the same clones. We use a clonal grass, *Festuca rubra*, as a model species. We propagated individual clones and used them in a transplant experiment across broad-scale temperature and precipitation gradients, simulating the projected direction of climate change in the region. Independent information on trait maxima and plasticities of the same clones was obtained by cultivating them in four growth chambers representing climate extremes. Plant survival was affected by interaction between plant traits and climate change, with both trait plasticities and maxima being important for adaptation to novel climates. Key traits include plasticity in extravaginal ramets, aboveground biomass, and osmotic potential. The direction of selection in response to a given climatic change detected in this study mostly contradicted the natural trait clines indicating that short-term selection pressure as identified here does not match long-term selection outcomes. Long-lived clonal species exposed to different climatic changes are subjected to consistent selection pressures on key traits, a necessary condition for adaptation to novel conditions. This points to evolutionary rescue as an important mechanism for dealing with climate change in these species. Our experimental approach may be applied also in other model systems broadening our understanding of evolutionary rescue. Such knowledge cannot be easily deduced from observing the existing field clines.

## Highlights

- Clonal plants are an excellent but underexploited model system for testing evolutionary hypotheses and mechanisms.
- Field reciprocal transplant experiments offer a powerful approach to study evolutionary rescue.
- We demonstrate that long-lived clonal plants may experience evolutionary rescue.
- Short-term trait selection does not always match long-term trait clines across climatic gradients.
- Both trait plasticities and trait maxima are important for adaptation to novel climates.
- Evolutionary rescue should be considered in future models of species adaptation to a changing climate.

## Introduction

Evolutionary rescue refers to the ability of a population to adapt to a changing environment from the standing genetic variation (Gomulkiewicz and Holt, 1995; Bell and Collins, 2008; Hufbauer et al., 2015). In addition to migration and phenotypic plasticity (Malcolm et al., 2002; Nicotra et al., 2010), evolutionary rescue is a key mechanism allowing species to adaptively respond to changing conditions (Bell, 2017). Evolutionary responses are potentially critical to ensuring population and species persistence under climatic changes of the rates and magnitudes we are seeing today (Diniz et al., 2019). The predicted rates of evolutionary response have, however, been suggested to be much slower than the predicted rates of climate change (Etterson and Shaw, 2001), although current empirical knowledge on species evolutionary responses and potentials is still limited (Diniz and Bini, 2019).

Research on the potential for evolutionary rescue includes mostly studies on evolution of resistance in microorganisms (reviewed in Bell, 2017, later e.g., Iriart et al., 2020). Empirical studies in long-lived species, such as plants, are mostly limited to resurrection experiments (Franks et al., 2008) attainable for annuals with persistent seed bank (Franks et al., 2007; Nevo et al., 2012; Thomann et al., 2015) and comparisons of populations from an inferred identical source exposed for longer periods to novel environments (using, e.g., introduced species, Lustenhouwer et al., 2018), which, however, do not allow causal understanding of the patterns observed.

Useful insights into trait selection by different climates in plants have been obtained using climate manipulation experiments (e.g., Avolio and Smith, 2013; Ravenscroft et al., 2014; Henn et al., 2018). The results of these studies are, however, limited by an unknown initial trait composition of the populations. This issue has been improved by Peterson et al. (2020) who explored selection on different *a priory* known ecotypes of *Mimulus guttatus*, all planted into a single location within a transplant experiment over two contrasting years and observed selection on these ecotypes. In our study, we build upon this general idea but extend it by using a clonal plant species as a model.

Clonal plants such as grasses are crucial components of many ecosystems and play a key role in their functioning (Gibson, 2009; Knappova et al., 2018). Understanding the functioning and adaptive potential of clonal plants is thus crucial for our ability to predict future ecosystem changes. These plants have many features that make them hard to study in an eco-evolutionary context (long-lived, large genomes, and often polyploids), but their clonality also offers opportunities as an experimental system similar to recombinant inbred lines in annuals and microorganisms. Using these plants allowed the application of a multipronged and replicated approach (i) exploring selection on continuous traits, (ii) replicating the experiments across multiple changed conditions with proper controls and spatial replicates, and (iii) combining field experiments with detailed lab essays to quantify trait variation and responses.

We make use of this advantage and demonstrate a novel approach to understand the potential for evolutionary rescue in long-lived species using a clonal grass, *Festuca rubra*, as a model. By propagating clonal plants in controlled settings, we got access to many replicate individuals (ramets) of the same genotype and at the same time cover the existing clonal variation. This material could be used for characterization and experimentation in natural and controlled environments, allowing us to explore selection of traits measured independent on the environment in which they currently grow. Specifically, we aimed to assess if traits assessed previously under controlled conditions (Münzbergová et al., 2017; Kosová et al., 2020) affect plant survival and growth under specific field conditions, and thus whether different trait combinations get selected under different climates. We also aimed to assess if the traits being selected under different climates correspond to trait clines of the genotypes naturally occurring in these specific conditions.

The study was done in a unique climatic grid established in western Norway allowing to separate the effects of temperature and precipitation on plant performance (Münzbergová and Hadincová, 2017; Münzbergová et al., 2017; Delnevo et al., 2018; Topper et al., 2018; Vandvik et al., 2020). Here, we used this climate grid to identify the effect of temperature, precipitation, and their interaction on possible trait selection under different climates. Exposing different populations to the same magnitude of climate shift but with different climatic starting points allowed us to assess the generality of the observed patterns (Vandvik et al., 2020). We compared the direction of selection detected in this study to natural trait clines occurring in the system and to plastic responses to the same climates simulated in growth chambers using data from previous studies (Münzbergová et al., 2017; Kosová et al., 2020).

Specifically, we asked the following questions: (1) Do changes in climatic conditions affect survival and growth of the transplanted genotypes? (2) Do populations exposed to specific changes in climate experience selection for specific trait values and trait plasticities, indicating potential for evolutionary rescue? (3) Do the conclusions differ depending on the fitness trait measured (survival or ramet production)? (4) Is there consistency between the short-term selection responses in the field, the general direction of field trait clines, and plastic responses to climate simulations under controlled conditions?

We hypothesized the following. (i) Because the plants are adapted to their local conditions, shift to warmer and moister conditions as well as their interaction will negatively affect survival and growth of all genotypes. (ii) Different changes in climatic conditions will select plants with different traits, indicating that populations of the species may be able to experience evolutionary rescue. (iii) Plant survival will be more dependent on plant traits than growth of the individuals that survived. (iv) The direction of the short-term trait selection will correspond to natural trait clines observed at localities indicating that short-term selection matches the long-term trends, and to plastic responses of the species to climate variation simulated in growth chambers.

## Methods

### Study System

We used *Festuca rubra* L. as a model species. *Festuca rubra* is a common perennial grass species of temperate, boreal, and alpine grasslands in Europe. The species may occur in different cytotypes, but we used only the most widespread hexaploid type in the experiment (Dirihan et al., 2016; Šurinová et al., 2019). It reproduces by seeds as well as vegetatively, producing intravaginal tillers and extravaginal tillers on rhizomes. *Festuca rubra* possesses considerable genetic variability both within and among populations and phenotypic plasticity (Herben et al., 2001; Münzbergová et al., 2017; Šurinová et al., 2019).

The experimental plants were collected across 12 sites constituting a natural climatic grid established in western Norway (for the SeedClim Grid, see Klanderud et al., 2015; Vandvik et al., 2020). It comprises 12 grassland localities representing three levels of summer temperature [the experiment was set up to achieve means of the four warmest months for individual locality types of ca. 6.5°C (alpine, ALP), 8.5°C (sub-alpine, SUB), and 10.5°C (boreal, BOR) combined with each of four levels of mean annual precipitation, ca. 600 (1), 1,300 (2), 2,000 (3), and 2,700 (4) mm; Meineri et al., 2014; Klanderud et al., 2015; Vandvik et al., 2020]. This climatic grid covers large part of conditions of natural occurrence of *F. rubra*. The target communities are grazed intermediate-rich meadows (Potentillo-Festucetum ovinae; G8 *sensu* Fremstad, 1997) occurring on southwest-facing (with the exception of one site, (BOR 3), which was exposed to the east), shallow slopes (5–20°) with a relatively base-rich bedrock. Sites were selected specifically to ensure that grazing regime and grazing history, bedrock, slope, aspect, and vegetation types are as similar as possible. The geographical distance between sites is on average 15 km and ranges from 0.65 km (BOR2 and SUB2) to 175 km (BOR1 and BOR4; Meineri et al., 2014).

### Plant Material

Münzbergová et al. (2017) collected 25 clones at each locality except for ALP2, where the species was not present. The plants were cultivated and propagated in the experimental garden of the Institute of Botany, Czech Academy of Sciences, Pruhonice, Czech Republic, since August 2014. Genetic analysis of the material (Šurinová et al., 2019) indicated that most of the clones represent unique genotypes (259 out of the 275 genotypes were unique genotypes, but for simplicity all the sample individuals are referred to as genotypes in the subsequent text). These clones have been used within the previous study (Münzbergová et al., 2017) in which each genotype was cultivated in four growth chambers representing the four climatic extremes of the climatic grid (i.e., driest and wettest combined with coldest and warmest). Within this experiment, we measured the total number of ramets, number of extravaginal ramets, plant height, aboveground and belowground biomass, and their ratio and rhizome weight. In addition, a parallel study (Kosová et al., 2020) measured the maximum quantum yield of primary PS II photochemistry (ϕP0; Stirbet and Govindjee, 2011) and osmotic potential (details of methods in Kosová et al., 2020). This resulted in nine different plant traits. We excluded rhizome weight from the final dataset as it was closely correlated with the proportion of extravaginal ramets. We also excluded data on belowground biomass as it was already used in the calculation of the ratio of above- to belowground biomass and was thus redundant with this variable and data on aboveground biomass. Finally, we excluded the information on maximum quantum yield of primary PS II photochemistry, as it did not have any information value in preliminary analyses. As a result, we finally worked with six different plant traits: plant height, number of ramets, proportion of extravaginal ramets, production of aboveground biomass, below:aboveground ratio, and osmotic potential.

These traits were measured for each genotype in four growth chambers representing the four climatic extremes of the natural climatic grid (Münzbergová et al., 2017). We used these values to express maximum trait value (as a measure of species maximum growth potential to grow across the environments), trait mean, and trait plasticity. Trait plasticity was expressed as (|trait_MAX_ − trait_MIN_|)/trait_MAX_, where trait_MAX_ and trait_MIN_ are respectively the maximal and minimal values of the trait measured on the same genotype across the four growth chambers (Valladares et al., 2000). As the mean values did not bring any additional insights compared to trait maxima (due to being largely correlated with the maxima), the means are not considered further. We preferred to use the maximal values rather than the means as the maxima reflect potential performance of the plants under optimal conditions, i.e., in any of the four growth chambers in which the specific genotype performs the best.

### Experimental Design

**Figure 1:**
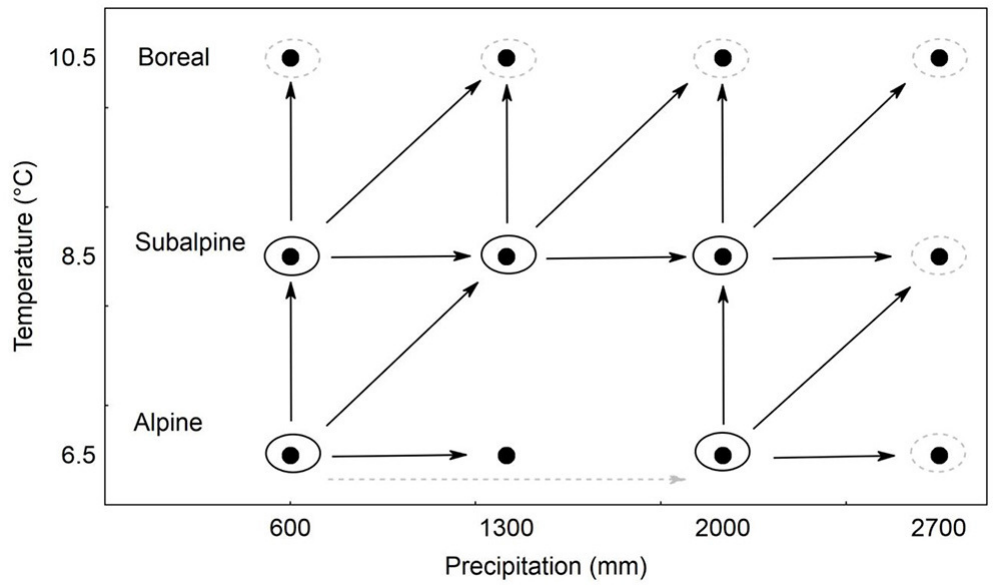
Design of the reciprocal transplant experiment across sites distributed along precipitation and temperature gradients in western Norway. Filled circles indicate the single localities. Arrows indicate the direction of the transplant. Large circles indicate populations planted to their home sites. Only transplants from the source populations encircled in black have been analyzed in this study. Transplants in gray served as controls but have not been used for the main analysis presented.

The climatic projection for western Norway is that the climate will become warmer and moister (Hanssen-Bauer et al., 2009). To study the species' ability to adapt to future conditions, we thus transplanted plants to warmer, moister, and warmer+moister sites along the climatic grid. In this way, we are simulating changes in mean climatic conditions experienced by the species. For the transplant experiment, we only used populations for which all these three shifts could be realized within the experimental grid (Figure 1). This resulted in five different original populations (Figure 1), as *F. rubra* was missing at one locality—ALP2. At each location (except ALP2), we also planted local individuals, which served as destination control (see below). Due to the missing destination control at ALP2, shift of the ALP1 population to moister conditions was done to ALP2 as well as ALP3 (Figure 1). We used 25 different genotypes from each population and planted each genotype in three replicates at each target locality (Figure 1). This resulted in 2,025 planted ramets in total. The data on plants that shifted from ALP1 to ALP3 were, however, not considered in the further analyses because we only wanted to compare plants unshifted to plants shifted by one step along the grid.

### Experimental Setup and Data Collection

We cut 10–20 young ramets from each of 25 genotypes per population and let them root in plastic cups in May 2015. When rooted, we transplanted selected ramets to multipots (with cells of 1 × 1 cm) and let them grow until mid-July 2015. We planted the transplant into the field in mid-July as this is the period when the growing season starts in the most extreme localities (i.e., all the snow is finally melted and the plants start growing). The number of planted ramets per genotype differed between populations and depended on the transplant scheme (see Figure 1). For each target site, we used three replicates of each genotype. Afterward, each of the ramets received a small colored plastic ring for future identification and the plants were transported to the experimental sites. At each site, we set up four to 18 transects divided into one to three blocks (depending on the shape of the available space suitable for planting at the locality, e.g., avoiding larger stones, shrubs, or depressions, and on the number of planted genotypes at the given locality). Vegetation at the selected location was cut to 5 cm height, and the single ramets were transplanted into the sward. Ramets were placed 10 cm apart within transects, and the transects were 10 cm apart. We created small disturbances, ~2 × 2 cm, when planting the ramets, to aid ramet establishment. Each ramet received a tag with a unique code.

The position of the specific ramets along the transects was set as follows. First, we randomized ramets from each source population separately for each replicate (i.e., three randomized sets per source population) and numbered them. We then combined first ramets from each source from the first replicate and planted them in random order. Afterward, second ramets from each source from the first replicate were planted in a random order, etc. We first planted all the ramets considered as the first replicate, followed by all the plants representing the second and then the third replicate. In this way, the positions of the ramets were randomized while ensuring that ramets of different origins followed one after each other (see Supporting Information 1 for illustration of the design). All plants were watered immediately after planting but did not receive any other treatment later.

We revisited the field sites at the end of July 2016 and 2017. At each occasion, we located each plant, recorded its survival, and counted the number of vegetative and generative ramets and their height for all living plants. However, as <1% of plants flowered, the data on generative ramets were not analyzed further but added to vegetative ramets into the variable “total number of ramets,” which were used in all analyses. In addition, we added plastic colored rings to all new ramets, to aid their detection next year. After recording all the ramets at the given locality, we cut all the vegetation including the transplants at 5 cm to simulate the historic management regime at the localities (low-intensity free-range grazing by domestic ungulates). While the animals are still grazing at the locality, the experimental plots have been fenced to avoid grazing damage.

### Data Analysis

To analyze the effect of plant traits (obtained in a previous growth chamber experiment; Münzbergová et al., 2017; Kosová et al., 2020) on species response to changing climate in the field experiment, we selected only clones from populations for which we had data on all the field transplant shifts (five populations planted to their home conditions and conditions warmer, moister, and warmer+moister, shift marked in black in Figure 1).

We tested how transplant survival and ramet number depend on standardized values (mean = 0, SD = 1) of plant traits (measured previously within a growth chamber experiment) and their interaction with temperature and precipitation change (coded as original/shifted) as fixed factors. The tests were done using a generalized mixed-effect model with genotype identity and target locality as random factors in the lme4 package (Bates et al., 2015) in R Development Core Team (2011). In this way, we first tested the effect of each trait separately. Afterward, we created a combined model containing all the separately significant factors in a single model. The dependent variables were transplant survival, assuming binomial distribution, and number of ramets, assuming Poisson distribution (done only for the living ramets). By calculating the relationship between transplant performance and standardized trait values, we in fact calculated selection gradients as suggested by Lande and Arnold (1983) and the data can thus be interpreted as the intensity of selection on the specific traits (see, e.g., Gomez-Gonzalez et al., 2011). We used the generalized mixed-effect model for this purpose due to the hierarchical nature of our data. Rolhauser et al. (2019) suggested that selection effects may not be linear, but rather quadratic. We thus also added the quadratic version of the traits into our model and tested if the models improved.

To calculate the strength of the effects, we calculated variance explained by the significant variables. As we used mixed-effect models, which do not allow straightforward calculation of residual variance (Kuznetsova et al., 2017), we expressed the variance out of total variance described by the whole model. These results are summarized in Supporting Information 2.

We separately analyzed data from 2016 and 2017. As the data from 2016 did not bring any additional insights to the later data, we only represent results for ramet survival until 2017 (i.e., including mortality in 2016) and ramet numbers in 2017. Over the 2 years, 52% of the transplanted ramets survived.

To summarize the results, we compared the observed direction of selection (i.e., the selection for specific trait values in this study) to natural field clines (i.e., effects of environment of plant origin detected in previous studies) of the same traits and changes of these traits under specific conditions in growth chambers as identified in previous studies (Münzbergová et al., 2017; Kosová et al., 2020). These data are based on the direction of the response of the specific traits and their plasticities to temperature and moisture of plant origin and their interaction and to temperature and moisture in the growth chambers and their interaction.

## Results

Transplant survival was significantly negatively affected by increased temperature but was independent of increased moisture and their interaction (Table 1). When tested separately for each of the five source populations, shift to higher temperature led to increased mortality in four of the five source populations (except for SUB2). In contrast, shift to moister conditions had a significant effect only in two source populations (SUB3 and ALP3). In both cases, ramet survival was higher in the moister than in the original localities. The interaction between change in temperature and change in moisture was never significant when tested for each population separately (*p* > 0.15 in all cases).

**Table 1:** The effects of trait plasticity and trait maxima (previously measured in growth chambers) in interaction with shift in temperature (T) and moisture (M) on ramet survival in the field experiment.

	**Plant trait**	**Shift in**	**Trait plasticity**				**Trait maxima**				
			***F***	***P***	**Selection**	**Cline**	***F***	***p***	**Selection**	**Cline**	**Gr. ch. plasticity**
Height:Ctemp	Plant height	T	1.27	0.239	n.s.	C↑W↓	1.07	0.315	n.s.	C↓W↑	C↓W↑
Height:Cmois		M	0.65	0.42	n.s.	D↑M↓	3.5	0.063*	n.s.	n.s.	D↑M↓
Height:Ctemp:Cmois		T x M	0.13	0.725	n.s.	n.s.	0.09	0.769	n.s.	CM↓,WM↑	CM↓
Ramet:Ctemp	Ramet no.	T	0.16	0.604	n.s.	n.s.	1.43	0.251	n.s.	n.s.	C↓W↑
Ramet:Cmois		M	1.3	0.249	n.s.	D↑M↓	0.48	0.483	n.s.	n.s.	D↑M↓
Ramet:Ctemp:Cmois		T x M	1.96	1.959	n.s.	n.s.	2.52	0.114	n.s.	n.s.	n.s.
Extra:Ctemp	Extravag. ramets	T	0.67	0.38	n.s.	n.s.	0.95	0.31	n.s.	n.s.	n.s.
Extra:Cmois		M	0.01	0.918	n.s.	n.s.	0.79	0.373	n.s.	n.s.	D↑M↓
Extra:Ctemp:Cmois		T x M	4.45	0.035*	CD↑, CM↓	n.s.	1.36	***0.04***	*CM↓WM↓*	CD↑WM↓	CD↑, CM↓
Above:Ctemp	Aboveg. biom.	T	0.67	0.453	n.s.	n.s.	0	0.964	n.s.	C↓W↑	C↓W↑
Above:Cmois		M	3.88	**0.05**	D↓	D↑M↓	0.39	0.537	n.s.	D↓M↑	D↑M↓
Above:Ctemp:Cmois		T x M	0.07	0.798	n.s.	n.s.	0.47	***0.007***	*CM↓WM↓*	CM↓WM↑	WD↑
Ratio:Ctemp	Root:shoot ratio	T	0.07	0.787	n.s.	n.s.	0.51	0.816	n.s.	n.s.	C↑W↓
Ratio:Cmois		M	0.02	0.898	n.s.	n.s.	1.94	*0.073*	n.s.	n.s.	D↑M↓
Ratio:Ctemp:Cmois		T x M	2.66	0.105	n.s.	n.s.	3.31	0.050*	WM↓	n.s.	n.s.
Osmotic:Ctemp	Osmotic potential	T	0.96	0.324	n.s.	C↓W↑	0.37	0.453	n.s.	n.s.	C↓W↑
Osmotic:Cmois		M	6.31	**0.009**	D↓, M↑	D↑M↓	4.72	**0.03**	M↑	n.s.	n.s.
Osmotic:Ctemp:Cmois		T x M	1.69	***0.011***	*WM↑*	n.s.	1.3	0.254	n.s.	n.s.	WM↑

Main effects of traits were never significant and are thus not shown. Ramet survival was significantly affected by T (F = 8.79, p = 0.004), but not by M (F = 1.84, p = 0.203) and its interaction with T (F = 0.45, p = 0.504). Variables marked by ^*^ are significant when all the previously significant variables have been included into a single model (jointly for trait plasticities and maxima). Arrows in the column Selection indicate direction of selection in a given environment (C-cold, W-warm, D-dry, M-moist). The original conditions are cold-dry as defined by our transplant design. Cline indicates direction of natural clines based on previous studies (effect of plant origin, Münzbergová et al., 2017; Kosová et al., 2020). Gr. ch. plasticity indicates plastic response to cultivation in different growth chambers based on previous studies as the clines. Values in bold are significant (p ≤ 0.05). Results which are underlined and in italics indicate significant effect after including a quadratic term into the model.

**Figure 2:**
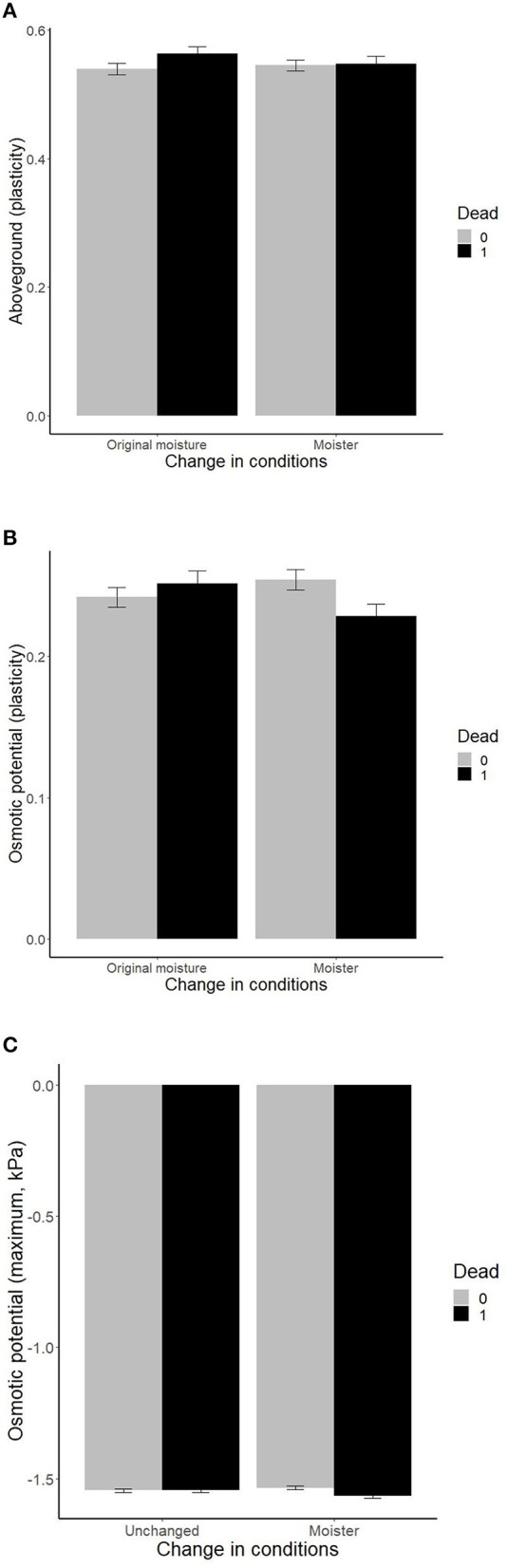
Effects of plant traits [**(A)** plasticity in aboveground biomass, **(B)** plasticity in osmotic potential, and **(C)** maximum values of osmotic potential] measured within previous growth chamber experiments (Münzbergová et al., 2017; Kosová et al., 2020) on survival of ramets within the field transplant experiment under different levels of moisture. The values represent mean ± SE.

While transplant survival was independent of the main effects of the growth chamber trait plasticities and maxima (*p* > 0.12 in all cases), the effects of traits interacted with changes in temperature, moisture, or their interaction (the change represents shift in the conditions experienced by the plants in the field; by accounting for plant source and genotype, we are comparing the effect of the shift in climate within each genotype, Table 1). Plants with high plasticity in aboveground biomass and osmotic potential suffered higher mortality under original moisture than plants with low plasticity, suggesting selection against high plasticity under ambient “home site” conditions (Figures 2A,B). Under increased moisture, ramet mortality was independent of aboveground biomass plasticity, and mortality was higher in ramets with lower plasticity in osmotic potential (Figures 2A,B). Increased moisture thus selected genotypes with higher plasticity in these two traits. In addition, plants with more negative values of maximum osmotic potential suffered higher mortality in increased moisture than plants with less negative values of maximum osmotic potential, but no significant effect was detected under original moisture (Figure 2C). No additional double interaction has been detected when adding quadratic versions of the traits (Table 1).

**Figure 3:**
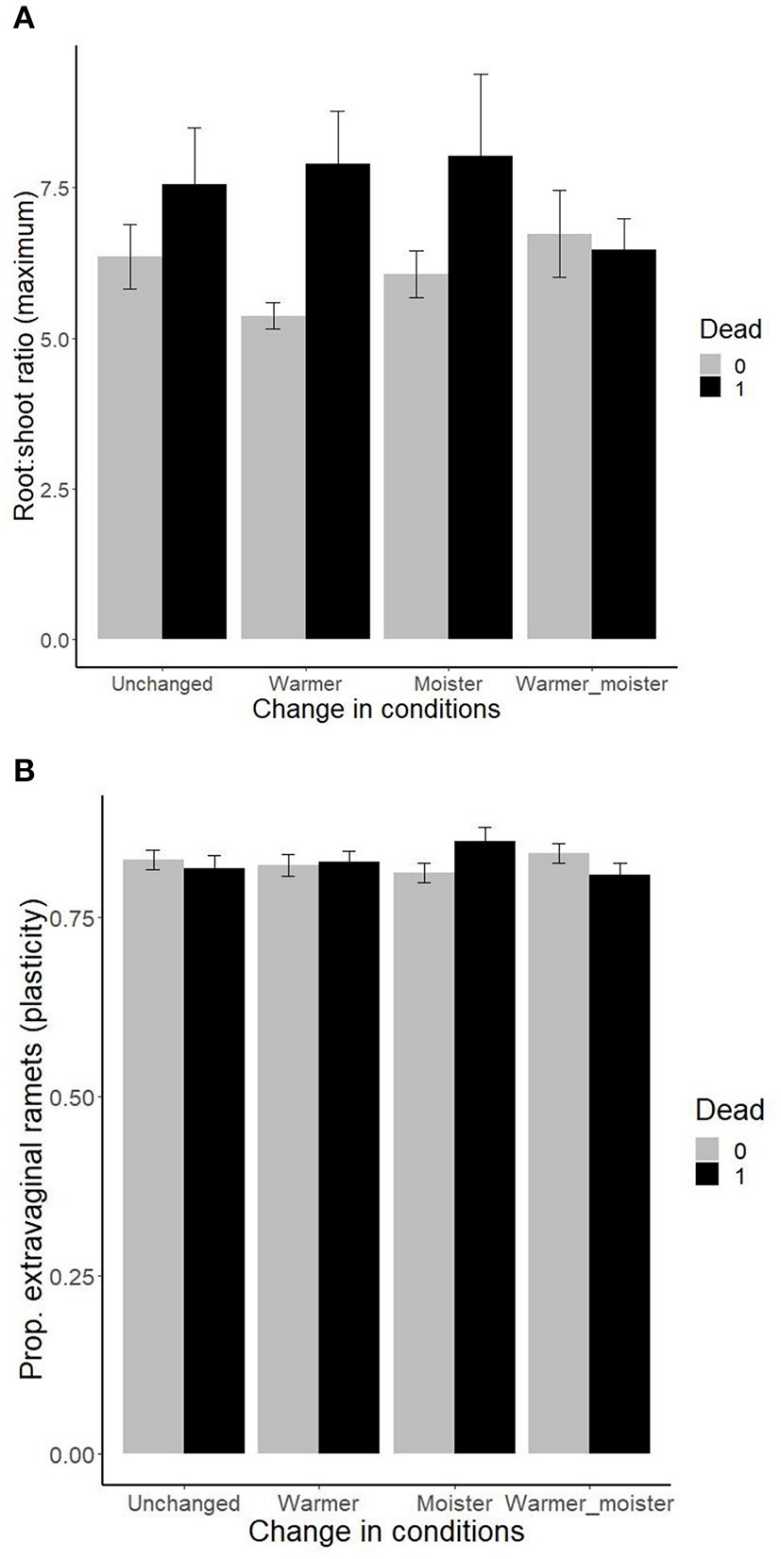
Effects of plant traits [**(A)** plasticity in root:shoot ratio and **(B)** plasticity in production of extravaginal ramets] measured within previous growth chamber experiments (Münzbergová et al., 2017; Kosová et al., 2020) on survival of ramets within the field transplant experiment under different levels of moisture and temperature. The values represent mean ± SE.

There were also two significant three-way interactions between the growth chamber plant traits, change in moisture, and change in temperature in their effect on plant survival. Plants with a higher maximum root:shoot ratio suffered higher mortality when both temperature and moisture were increased, but not when none or both climatic parameters were changed (Figure 3A). Plants with lower plasticity in proportion of extravaginal ramets showed increased mortality under original conditions and under warmer+moister conditions. At the same time, plants with higher plasticity in proportion of extravaginal ramets showed increased mortality under moister conditions, and there was no difference in survival of the ramets under warmer conditions (Figure 3B). When including the quadratic version of the traits, three more significant triple interactions (i.e., trait, change in moisture, and change in temperature) have been detected, namely, for plasticity in osmotic potential, and maximum in proportion of extravaginal ramets and aboveground biomass (Table 1, Supporting Informations 3A–C). Variance explained by single significant variables of the total variation explained by the models ranged between 17 and 32% and is shown in Supporting Information 2.

In contrast to the transplant survival, the ramet number of the plants grown in the field transplant experiment was independent of any effect of climate change, plant traits, and their interactions (*p* > 0.058 in all cases). The results with ramet number as the dependent variable are thus not presented further.

Trait selection trends identified in this study were not consistent with field clines for the same traits based on previous studies (Table 1). The only partial exception was selection for a higher proportion of extravaginal ramets in a cold-dry environment, but the same selection has been observed in all the other environments except cold-moist. We also did not detect any consistent patterns among the trait selection detected here and trait plastic response to the same environment simulated within growth chambers as observed previously (Table 1). In most cases, we either detected significant selection in this study, and no significant effects in the previous studies or vice versa. In two cases, we got significant results in both the current and previous studies, but with opposite directions. This happened for plasticity in aboveground biomass and osmotic potential. Here drier, i.e., original, conditions selected plants with lower plasticity, while plants from drier conditions had higher plasticity values.

## Discussion

A key finding of our study is that mortality of genotypes grown under different conditions in the field depends on their traits, measured previously within growth chambers, with specific climatic conditions (mainly moisture) exerting differential selection pressure on different traits. In several cases, the selection pressure depended on the interaction between temperature and moisture, indicating that these variables interact in their selection pressure on the plants. Different climatic conditions thus select genotypes with specific traits, possibly allowing the populations to adapt to local condition within a single clonal generation. In other words, the section acting on the population has the potential to select genotypes with specific sets of traits leading to shift in trait means and their variation making the whole population better adapted to the local conditions. This indicates that even long-lived clonal plant species as studied here have the potential to experience the evolutionary rescue.

### Evolutionary Rescue

Significant trait selection due to changes in climate as found in our study has been previously confirmed mainly in short-lived species. For example, using so-called resurrection experiments (Franks et al., 2008) comparing plants grown from old and new seed material (Franks et al., 2007; Nevo et al., 2012; Thomann et al., 2015) demonstrated significant shifts in flowering phenology and flower morphology due to recent climate warming and land-use changes suggesting that plants have undergone significant trait selection over a few decades. Similar patterns have also been detected in studies of Avolio and Smith (2013) and Ravenscroft et al. (2014), demonstrating significant differences in traits (estimated under standardized conditions) in plants that had been exposed to different climate manipulations. Their results are, however, limited by the fact that the initial trait composition of the plots was unknown. Our results are thus first to demonstrate the potential for evolutionary rescue in long-lived perennial plants, using real field conditions but strictly controlling for the initial trait composition of the material exposed to the selection.

Both trait maxima and trait plasticities were differentially selected under different conditions. Specifically, plants that shifted to moister conditions experienced strong selection toward increased plasticity in osmotic potential, while plants in drier (i.e., original) conditions experienced the opposite selection with the strongest positive selection acting in warmer-moister conditions. Drier conditions also selected against plasticity in aboveground biomass. In contrast, higher plasticity in proportion of extravaginal ramets was selected in cold-dry (i.e., original) conditions as well as in warm-moist conditions (i.e., expected climate change), but not in the other two climate combinations (cold-moist, warm-dry). High trait plasticity is often viewed as a positive species trait allowing species to occupy wider environmental niches (Bradshaw, 1965; Baker, 1974; Murray et al., 2002; Hofmann and Todgham, 2010). However, high plasticity in a fitness-related trait may also indicate that the genotype is unable to maintain its function under stressful conditions (Dostal et al., 2016). In addition, maintenance of the ability of being plastic may be costly reducing the possible advantages of plasticity under certain conditions (DeWitt et al., 1998; van Kleunen and Fischer, 2007; Auld et al., 2010).

We argue that plasticity of osmotic potential and proportion of extravaginal ramets should confer a selective advantage under extreme conditions (in our system, the extreme conditions seem to be represented by high moisture for osmotic potential and warm-wet and cold-dry conditions for proportion of extravaginal ramets). Osmotic potential has been shown to have a high potential to respond to actual conditions in some systems including ours (e.g., Knutzen et al., 2015; Kosová et al., 2020, but not in other systems, Pratt and Mooney, 2013) and may allow the plants to maintain their fitness under stressful conditions represented by various moisture and temperature combinations (Bartlett et al., 2014). Similarly, switch between production of extravaginal and intravaginal ramets has been shown to be highly plastic allowing species to respond to stand heterogeneity and switch between the guerrilla and phalanx growth strategy (Herben et al., 2001; Ye et al., 2006; Skalova, 2010; Münzbergová et al., 2017). Stand heterogeneity is largely affected by performance of other species in the community and is thus likely to reflect changes in climatic conditions, such as increased competition under warmer and moister climates (Klanderud et al., 2015, 2017; Olsen et al., 2016; Vandvik et al., 2020). In case of aboveground biomass, a trait which is likely related to fitness (e.g., Mason et al., 2017), high plasticity may indicate inability to maintain fitness under adverse conditions. Selection against plasticity in this trait may thus indicate selection against plants without physiological adaptations to cope with local conditions. In general, the observed selection against plasticity in some cases indicates that the plasticity studied here was not always adaptive. This is in line with the general expectation that plasticity may and may not have an adaptive value (van Kleunen and Fischer, 2005). The adaptivity of plasticity differs among traits and depends on the extent of spatial and temporal variation in the system and thus its predictability (Cohen, 1966; Miner et al., 2005).

In terms of maximum trait values, warmer-moister conditions selected plants with a lower root:shoot ratio and higher aboveground biomass. This may reflect increased competition for light in warmer-moister conditions as suggested by the stress-gradient hypothesis (Callaway et al., 2002; He et al., 2013; Klanderud et al., 2017), where plants are forced to invest into aboveground biomass (Shao et al., 2018) and plants with genetic predisposition to do so are favored. Moister conditions also selected plants with less negative osmotic potential. High negative osmotic potential allows plants to acquire water in very dry sites (Bartlett et al., 2014), and selection against this trait may thus serve as a protection against water excess. Note that our wettest sites have 2,700 mm of annual rainfall, so excess of water may be a real problem at these sites. Selection against a high proportion of extravaginal ramets in a cold-moist environment is in line with selection against high plasticity in this trait as discussed above.

Detection of significant trait selection in climatic conditions representing expected future climates (i.e., moister, warmer or warmer+moister) is in line with the expectation of the evolutionary rescue in these populations. However, several results indicated also significant selection in the extant cold-dry, i.e., original, conditions. This is surprising, because the selection operates on plants naturally occurring at the localities. This may be explained by the fact that the localities have already experienced changes in the conditions in the past decades such as increased frequency of extreme drought events (Lindner et al., 2010; IPCC, 2014) and the conditions have become unsuitable for plants with certain traits. This finding is in line with Peterson et al. (2020), indicating that the current distribution of species traits may lag behind the changes in the distribution of suitable habitat conditions leading to so-called adaptational lag (see also Gray et al., 2011; Wilczek et al., 2014; McGraw et al., 2015). The plants with unsuitable traits may still survive at the localities because they are very long-lived and clonally reproducing (Harberd, 1961; de Witte and Stocklin, 2010) and the most critical is the survival over the 1st years, as shown here. A similar effect has also been repeatedly observed at the species level in many perennial species and is known as extinction debt (Kuussaari et al., 2009; Krauss et al., 2010; Dullinger et al., 2012; Plue et al., 2017; Mairal et al., 2018).

The strong selection acting in the populations may also indicate that over time, individuals with certain traits will be lost, leading to reduced variation in the populations and thus loss of the ability to adapt to changes in opposite direction (Orive et al., 2019). This, however, contrasts with the fact that the existing populations have high variation both in terms of genetic variation (Šurinová et al., 2019) and in term of traits (Stojanova et al., 2018, 2019). This contradiction can be explained by between-year variation in the direction of selection (Ehrlén and Münzbergová, 2009; Kulbaba et al., 2019; Ehrlen and Valdes, 2020; Peniston et al., 2020) as the climate of the sites indeed shows high interannual variation (Vandvik et al., 2020). The effects of this variation are likely stronger in species with long-lived stages (aboveground or in seed bank) containing genotypes favored in different times (Vandvik et al., 2016; Plue et al., 2017; Topper et al., 2018).

The potential for evolutionary rescue detected in the current study provides unique evidence of the ability to maintain genotypes favored under various climatic conditions in clonal species. This is in line with a recent study, suggesting such a potential in corals (Rinkevich, 2019), representing a unique case of clonality in animals (Jackson and Coates, 1986). The high variation in our results, however, also indicates that the success of the evolutionary rescue in the species cannot be easily predicted. This may be attributed to the clonal nature of the species, as clonality may weaken the potential of evolutionary rescue in continually changing conditions (Orive et al., 2019). The high variation may also be given by context dependencies across landscapes, as different specific environmental conditions and changes may select for different specific traits and trait combinations (Vandvik et al., 2020). Well-replicated studies, such as this one, are needed to find generalities in these patterns.

It may be argued that our study is relatively short-term and we thus observed selection acting in a particular year in a set of specific weather conditions. While this is indeed true, the selection observed leading to mortality of individuals with specific sets of traits suggests that such a selection may lead to changes in genotypic composition of the populations, leading to evolutionary changes in these populations. The implications of our study in terms of evolutionary changes in the populations are even stronger as the effects observed were solely effects on survival rather than growth, thus leading to real elimination of the unsuitable genotypes. This is also in line with Fridley (2017), indicating that majority of energetic costs of a plant are invested into survival rather than growth, so plants differentially adapted to a given environment are more likely to differ in their survival than in their growth.

While we found a range of significant interactions between specific traits and change in climates, it needs to be noted that the differences in the trait values between surviving and dead plants are often quite small. In addition, our significant variables only form between one quarter and one third of the variance captured by our models. All this indicates that the selection pressure is not very high. However, the differences in the values between dead and surviving plants are in the same order of magnitude as the differences in the trait values among different original climates and climates of cultivation detected in the previous studies (Münzbergová et al., 2017; Kosová et al., 2020). This thus indicates that the selection detected in this study may lead to a shift in the traits within the range commonly observed in the field.

### Correspondence Between Short-Term Selection and Natural Field Clines

In contrast to expectations, we did not find a single occasion of correspondence between selection detected here and natural field clines detected in a previous study. In two cases (trait plasticity in aboveground biomass and osmotic potential in interaction with moisture, Table 1), we instead detected patterns going into the opposite direction. This contrasts to result of Dunne et al. (2004), indicating that responses to short-term climate manipulation correspond to natural environmental clines. This contrasting pattern indicates that current short- and past long-term selection pressures may go in opposite directions (similarly Saleska et al., 2002). In other words, the selection differs depending on whether we study selection by current weather over 1 or 2 years vs. by climate representing the long-term weather regime. Deducing the direction of selection from patterns detected in the field, as done in numerous previous gradient studies including ours (e.g., Merila and Hendry, 2014; Stojanova et al., 2018, 2019), may thus be misleading. More studies exploring the selection in action as done here are thus required to understand the real patterns of species responses to novel climates. Additional comparisons of short- and long-term selection pressures are required to elucidate species evolutionary responses to global climate change (e.g., O'Gorman et al., 2014).

Despite strong significant effects of conditions of plant cultivation (growth chambers) on the various traits in previous studies (Münzbergová et al., 2017; Kosová et al., 2020), we also did not find any correspondence between phenotypic plasticity observed in the growth chamber and trait selection observed in this study. Knowledge on trait plasticity in controlled conditions thus does not provide any insights into the potential selection pressure in natural conditions. This may be explained by the multivariate nature of the selection pressures in natural conditions compared to the simplified settings in the growth chambers.

### Effect of Changing Conditions on Plant Survival and Growth

Increased temperature, but not moisture or its interaction with temperature, negatively affected survival of the transplants in the field. This is in line with the conclusions of Hansen and Turner (2019) who state that temperature is a key factor affecting plant survival. As for many other gradient studies, they could not empirically separate the effects of increased temperature from the effects of decreased moisture, however. Our experiment is thus unique in demonstrating that temperature, but not moisture, is a key determinant of plant survival under climate change. Further, our replicated design allowed us to test for, and establish, that effects of increased temperature were independent of the extant values of temperature in the different sites, as the effect was significant and in the same direction in four out of five source populations tested (with different temperature in each source population, Figure 1), with the nonsignificant effects being detected in one intermediate population. This indicates that the effects are largely consistent across broad bioclimatic gradients.

The lack of any significant interaction between temperature and moisture, both in the total dataset and when tested separately for each source population, contrasts with the results of previous studies from the same system, demonstrating that both original and target moisture and temperature interact with each other to affect various species traits (Münzbergová et al., 2017) and population (Olsen et al., 2016) and community dynamics (Klanderud et al., 2015; Vandvik et al., 2020). Some of the previous interactions have been detected in simulated growth chamber conditions (e.g., Münzbergová et al., 2017) where the effects of climate reflect the mean conditions at the localities and are unaffected by between-year variation, the effects of which may differ among different sites. However, also previous field studies detected such an interaction (Klanderud et al., 2015; Vandvik et al., 2020). As they often worked with established species and their performance, their results still rather reflected means of the conditions across multiple years, while our young transplants were likely much more responsive to the actually conditions of the given years.

The lack of moisture effects and possibly also of the temperature and moisture interaction on transplant survival is possibly also linked to the fact that our wetness gradient goes from wet to very wet and our plants thus did not suffer any water stress. This contrasts to most other studies on the effects of changing moisture exposing plants to extremely dry conditions (e.g., Groves and Brudvig, 2019). Thanks to this, the effects of moisture are often nonlinear (Klanderud et al., 2017) and assuming the same effects from different starting points within the grid is thus unjustified. It is also possible that the effects of moisture at the locality were masked by the effects of microtopography on microclimate (e.g., Scherrer and Korner, 2010; Hansen and Turner, 2019), an effect that was not explicitly tested in our study.

## Conclusions

Conditions to which the plants were shifted affected the survival of transplanted ramets, and the survival success depended on plant traits. This indicates that populations exposed to novel climates (in our study represented by specific weather conditions in the novel locations over 2 years) can experience directional selection toward optimal traits leading to potential evolutionary rescue.

In contrast, we did not find any significant selection on specific traits due to climate change when using data on growth of the ramets which survived. This indicates that the key selection happens at the level of ramet survival and not at the level of subsequent growth. Early stages of plant establishment thus play a crucial role in species adaptation to novel conditions (Guittar et al., 2020). Overall, the results suggest that the ability of rapid adaptation leading to potential evolutionary rescue should be acknowledged as a potentially important mechanism allowing long-lived clonal plants to respond to climate change.

## Data Availability Statement

The raw data supporting the conclusions of this article will be made available by the authors, without undue reservation.

## Author Contributions

ZM and VH conceived the ideas, designed methodology, and collected the data. VV provided the system to conduct the experiment. ZM analyzed the data and led the writing of the manuscript. All authors contributed critically to the drafts and gave final approval for publication.

## Conflict of Interest

The authors declare that the research was conducted in the absence of any commercial or financial relationships that could be construed as a potential conflict of interest.

## References

[B1] Auld J. R. Agrawal A. A. Relyea R. A. (2010). Re-evaluating the costs and limits of adaptive phenotypic plasticity. Proc. Royal Soc. B Biol. Sci. 277, 503–511. 10.1098/rspb.2009.1355 19846457 PMC2842679

[B2] Avolio M. L. Smith M. D. (2013). Mechanisms of selection: phenotypic differences among genotypes explain patterns of selection in a dominant species. Ecology 94, 953–965. 10.1890/12-1119.1

[B3] Baker H. G. (1974). The evolution of weeds. Ann Rev Ecol Systemat. 5, 1–24. 10.1146/annurev.es.05.110174.000245

[B4] Bartlett M. K. Zhang Y. Kreidler N. Sun S. W. Ardy R. Cao K. F.. (2014). Global analysis of plasticity in turgor loss point, a key drought tolerance trait. Ecol. Lett. 17, 1580–1590. 10.1111/ele.12374 25327976

[B5] Bates D. Machler M. Bolker B. M. Walker S. C. (2015). Fitting linear mixed-effects models using lme4. J. Statist. Softw. 67, 1–48. 10.18637/jss.v067.i01

[B6] Bell G. (2017). Evolutionary rescue. Ann. Rev. Ecol. Evol. Systemat. 48, 605–627. 10.1146/annurev-ecolsys-110316-023011

[B7] Bell G. Collins S. (2008). Adaptation, extinction and global change. Evol. Appl. 1, 3–16. 10.1111/j.1752-4571.2007.00011.x 25567487 PMC3352406

[B8] Bradshaw A. D. (1965). Evolutionary significance of phenotypic plasticity in plants. Adv. Genet. 13, 115–155. 10.1016/S0065-2660(08)60048-6

[B9] Callaway R. M. Brooker R. W. Choler P. Kikvidze Z. Lortie C. J. Michalet R.. (2002). Positive interactions among alpine plants increase with stress. Nature 417, 844–848. 10.1038/nature00812 12075350

[B10] Cohen D. (1966). Optimizing reproduction in a randomly varying environment. J. Theoret. Biol. 12, 119–129. 10.1016/0022-5193(66)90188-3 6015423

[B11] de Witte L. C. Stocklin J. (2010). Longevity of clonal plants: why it matters and how to measure it. Ann. Bot. 106, 859–870. 10.1093/aob/mcq191 20880935 PMC2990663

[B12] Delnevo N. Petraglia A. Carbognani M. Vandvik V. Halbritter A. H. (2018). Plastic and genetic responses to shifts in snowmelt time affects the reproductive phenology and growth of *Ranunculus acris*. Perspectiv. Plant Ecol. Evol. Systemat. 30, 62–70. 10.1016/j.ppees.2017.07.005

[B13] DeWitt T. J. Sih A. Wilson D. S. (1998). Costs and limits of phenotypic plasticity. Trends Ecol. Evol. 13, 77–81. 10.1016/S0169-5347(97)01274-3 21238209

[B14] Diniz J. A. F. Bini L. M. (2019). Will life find a way out? Evolutionary rescue and Darwinian adaptation to climate change. Perspectiv. Ecol. Conserv. 17, 117–121. 10.1016/j.pecon.2019.06.001

[B15] Diniz J. A. F. Souza K. S. Bini L. M. Loyola R. Dobrovolski R. Rodrigues J. F. M.. (2019). A macroecological approach to evolutionary rescue and adaptation to climate change. Ecography 42, 1124–1141. 10.1111/ecog.04264

[B16] Dirihan S. Helander M. Vare H. Gundel P. E. Garibaldi L. A. Irisarri J. G. N.. (2016). Geographic variation in *Festuca rubra* L. ploidy levels and systemic fungal endophyte frequencies. PLoS ONE 11:166264. 10.1371/journal.pone.0166264 27846291 PMC5112939

[B17] Dostal P. Fischer M. Prati D. (2016). Phenotypic plasticity is a negative, though weak, predictor of the commonness of 105 grassland species. Glob. Ecol. Biogeogr. 25, 464–474. 10.1111/geb.12429

[B18] Dullinger S. Gattringer A. Thuiller W. Moser D. Zimmermann N. E. Guisan A.. (2012). Extinction debt of high-mountain plants under twenty-first-century climate change. Nat. Climate Change 2, 619–622. 10.1038/nclimate1514

[B19] Dunne J. A. Saleska S. R. Fischer M. L. Harte J. (2004). Integrating experimental and gradient methods in ecological climate change research. Ecology 85, 904–916. 10.1890/03-8003

[B20] Ehrlén J. Münzbergová Z. (2009). Timing of flowering: opposed selection on different fitness components and trait covariation. Am. Natural. 173, 819–830. 10.1086/598492 19335224

[B21] Ehrlen J. Valdes A. (2020). Climate drives among-year variation in natural selection on flowering time. Ecol. Lett. 23, 653–662. 10.1111/ele.13468 31994327

[B22] Etterson J. R. Shaw R. G. (2001). Constraint to adaptive evolution in response to global warming. Science 294, 151–154. 10.1126/science.1063656 11588260

[B23] Franks S. J. Avise J. C. Bradshaw W. E. Conner J. K. Etterson J. R. Mazer S. J.. (2008). The resurrection initiative: storing ancestral genotypes to capture evolution in action. Bioscience 58, 870–873. 10.1641/B580913

[B24] Franks S. J. Sim S. Weis A. E. (2007). Rapid evolution of flowering time by an annual plant in response to a climate fluctuation. Proc. Natl. Acad. Sci. U. S. A. 104, 1278–1282. 10.1073/pnas.0608379104 17220273 PMC1783115

[B25] Fremstad E. (1997). Vegetasjonstyper i Norge. NINA Temahefte. 12, 1–279.

[B26] Fridley J. D. (2017). Plant energetics and the synthesis of population and ecosystem ecology. J. Ecol. 105, 95–110. 10.1111/1365-2745.12693

[B27] Gibson D. J. (2009). Grasses and Grassland Ecology. Oxford: Oxford University Press.

[B28] Gomez-Gonzalez S. Torres-Diaz C. Bustos-Schindler C. Gianoli E. (2011). Anthropogenic fire drives the evolution of seed traits. Proc. Natl. Acad. Sci. U. S. A. 108, 18743–18747. 10.1073/pnas.1108863108 22065739 PMC3219139

[B29] Gomulkiewicz R. Holt R. D. (1995). When does evolution by natural selection prevent extinction. Evolution 49, 201–207. 10.1111/j.1558-5646.1995.tb05971.x 28593677

[B30] Gray L. K. Gylander T. Mbogga M. S. Chen P. Y. Hamann A. (2011). Assisted migration to address climate change: recommendations for aspen reforestation in western Canada. Ecol. Appl. 21, 1591–1603. 10.1890/10-1054.1 21830704

[B31] Groves A. M. Brudvig L. A. (2019). Interannual variation in precipitation and other planting conditions impacts seedling establishment in sown plant communities. Restorat. Ecol. 27, 128–137. 10.1111/rec.12708

[B32] Guittar J. Goldberg D. Klanderud K. Berge A. Boixaderas M. R. Meineri E.. (2020). Quantifying the roles of seed dispersal, filtering, and climate on regional patterns of grassland biodiversity. Ecology 101:3061. 10.1002/ecy.3061 32239491

[B33] Hansen W. D. Turner M. G. (2019). Origins of abrupt change? Postfire subalpine conifer regeneration declines nonlinearly with warming and drying. Ecol. Monogr. 89:21. 10.1002/ecm.1340

[B34] Hanssen-Bauer I. Drange H. Førland E. J. Roald L. A. Børsheim K. Y. Hisdal H.. (2009). Klima i Norge 2100 Bakgrunnsmateriale til NOU Klimatilpassing (Climate in Norway 2100 Background Material to the NOU Climate Adaption). Oslo: Norsk klimasenter.

[B35] Harberd D. (1961). Observations on population struture and longevity of *Festuca rubra* L. New Phytol. 60, 184–206. 10.1111/j.1469-8137.1961.tb06251.x

[B36] He Q. Bertness M. D. Altieri A. H. (2013). Global shifts towards positive species interactions with increasing environmental stress. Ecol. Lett. 16, 695–706. 10.1111/ele.12080 23363430

[B37] Henn J. J. Buzzard V. Enquist B. J. Halbritter A. H. Klanderuds K. Maitner B. S.. (2018). Intraspecific trait variation and phenotypic plasticity mediate alpine plant species response to climate change. Front. Plant Sci. 9:11. 10.3389/fpls.2018.01548 30483276 PMC6243391

[B38] Herben T. Krahulec F. Hadincova V. Pechackova S. (2001). Clone-specific response of *Festuca rubra* to natural variation in biomass and species composition of neighbours. Oikos 95, 43–52. 10.1034/j.1600-0706.2001.950105.x

[B39] Hofmann G. E. Todgham A. E. (2010). Living in the now: physiological mechanisms to tolerate a rapidly changing environment. Ann. Rev. Physiol. 2010, 127–145. 10.1146/annurev-physiol-021909-135900 20148670

[B40] Hufbauer R. A. Szucs M. Kasyon E. Youngberg C. Koontz M. J. Richards C.. (2015). Three types of rescue can avert extinction in a changing environment. Proc. Natl. Acad. Sci. U. S. A. 112, 10557–10562. 10.1073/pnas.1504732112 26240320 PMC4547288

[B41] IPCC (2014). Climate change 2014: Synthesis report.

[B42] Iriart V. Baucom R. S. Ashman T. L. (2020). Herbicides as anthropogenic drivers of eco-evo feedbacks in plant communities at the agro-ecological interface. Mol. Ecol. 16:15510. 10.1111/mec.15510 32542840

[B43] Jackson J. B. C. Coates A. G. (1986). Life-cycles and evolution of clonal (modular) animals. Philos. Trans. Royal Soc. B Biol. Sci. 313, 7–22. 10.1098/rstb.1986.0022

[B44] Klanderud K. Meineri E. Topper J. Michel P. Vandvik V. (2017). Biotic interaction effects on seedling recruitment along bioclimatic gradients: testing the stress-gradient hypothesis. J. Vegetat. Sci. 28, 347–356. 10.1111/jvs.12495

[B45] Klanderud K. Vandvik V. Goldberg D. (2015). The importance of biotic vs. abiotic drivers of local plant community composition along regional bioclimatic gradients. PLoS ONE 10:e0130205. 10.1371/journal.pone.0130205 26091266 PMC4474800

[B46] Knappova J. Zidlicka D. Kadlec T. Knapp M. Haisel D. Hadincova V.. (2018). Population differentiation related to climate of origin affects the intensity of plant-herbivore interactions in a clonal grass. Basic Appl. Ecol. 28, 76–86. 10.1016/j.baae.2018.02.011

[B47] Knutzen F. Meier I. C. Leuschner C. (2015). Does reduced precipitation trigger physiological and morphological drought adaptations in European beech (*Fagus sylvatica* L.)? Comparing provenances across a precipitation gradient. Tree Physiol. 35, 949–963. 10.1093/treephys/tpv057 26209617

[B48] Kosová V. Hájek T. Hadincová V. Münzbergová Z. (2020). Ecophysiological traits of a clonal grass in its climate change response. bioRxiv 2020:864827. 10.1101/864827

[B49] Krauss J. Bommarco R. Guardiola M. Heikkinen R. K. Helm A. Kuussaari M.. (2010). Habitat fragmentation causes immediate and time-delayed biodiversity loss at different trophic levels. Ecol. Lett. 13, 597–605. 10.1111/j.1461-0248.2010.01457.x 20337698 PMC2871172

[B50] Kulbaba M. W. Sheth S. N. Pain R. E. Eckhart V. M. Shaw R. G. (2019). Additive genetic variance for lifetime fitness and the capacity for adaptation in an annual plant. Evolution 73, 1746–1758. 10.1111/evo.13830 31432512

[B51] Kuussaari M. Bommarco R. Heikkinen R. K. Helm A. Krauss J. Lindborg R.. (2009). Extinction debt: a challenge for biodiversity conservation. Trends Ecol. Evol. 24, 564–571. 10.1016/j.tree.2009.04.011 19665254

[B52] Kuznetsova A. Brockhoff P. B. Christensen R. H. B. (2017). lmerTest package: tests in linear mixed effects models. J. Statist. Softw. 82, 1–26. 10.18637/jss.v082.i13

[B53] Lande R. Arnold S. J. (1983). The measurement of selection on correlated characters. Evolution 37, 1210–1226. 10.1111/j.1558-5646.1983.tb00236.x 28556011

[B54] Lindner M. Maroschek M. Netherer S. Kremer A. Barbati A. Garcia-Gonzalo J.. (2010). Climate change impacts, adaptive capacity, and vulnerability of European forest ecosystems. Forest Ecol. Manag. 259, 698–709. 10.1016/j.foreco.2009.09.023

[B55] Lustenhouwer N. Wilschut R. A. Williams J. L. van der Putten W. H. Levine J. M. (2018). Rapid evolution of phenology during range expansion with recent climate change. Glob. Change Biol. 24, E534–E544. 10.1111/gcb.13947 29044944

[B56] Mairal M. Caujape-Castells J. Pellissier L. Jaen-Molina R. Alvarez N. Heuertz M.. (2018). A tale of two forests: ongoing aridification drives population decline and genetic diversity loss at continental scale in Afro-Macaronesian evergreen-forest archipelago endemics. Ann. Bot. 122, 1005–1017. 10.1093/aob/mcy107 29905771 PMC6266103

[B57] Malcolm J. R. Markham A. Neilson R. P. Garaci M. (2002). Estimated migration rates under scenarios of global climate change. J. Biogeogr. 29, 835–849. 10.1046/j.1365-2699.2002.00702.x

[B58] Mason C. M. Goolsby E. W. Davis K. E. Bullock D. V. Donovan L. A. (2017). Importance of whole-plant biomass allocation and reproductive timing to habitat differentiation across the North American sunflowers. Ann. Bot. 119, 1131–1142. 10.1093/aob/mcx002 28203721 PMC5604586

[B59] McGraw J. B. Turner J. B. Souther S. Bennington C. C. Vavrek M. C. Shaver G. R.. (2015). Northward displacement of optimal climate conditions for ecotypes of *Eriophorum vaginatum* L. across a latitudinal gradient in Alaska. Glob. Change Biol. 21, 3827–3835. 10.1111/gcb.12991 26033529

[B60] Meineri E. Skarpaas O. Spindelbock J. Bargmann T. Vandvik V. (2014). Direct and size-dependent effects of climate on flowering performance in alpine and lowland herbaceous species. J. Veg. Sci. 25, 275–286. 10.1111/jvs.12062

[B61] Merila J. Hendry A. P. (2014). Climate change, adaptation, and phenotypic plasticity: the problem and the evidence. Evolution. Appl. 7, 1–14. 10.1111/eva.12137 24454544 PMC3894893

[B62] Miner B. G. Sultan S. E. Morgan S. G. Padilla D. K. Relyea R. A. (2005). Ecological consequences of phenotypic plasticity. Trends Ecol. Evol. 20, 685–692. 10.1016/j.tree.2005.08.002 16701458

[B63] Münzbergová Z. Hadincová V. (2017). Transgenerational plasticity as an important mechanism affecting response of clonal species to changing climate. Ecol. Evol. 7, 5236–5247. 10.1002/ece3.3105 28770062 PMC5528211

[B64] Münzbergová Z. Hadincová V. Skálová H. Vandvik V. (2017). Genetic differentiation and plasticity interact along temperature and precipitation gradients to determine plant performance under climate change. J. Ecol. 105, 1358–1373. 10.1111/1365-2745.12762

[B65] Murray B. R. Thrall P. H. Gill A. M. Nicotra A. B. (2002). How plant life-history and ecological traits relate to species rarity and commonness at varying spatial scales. Austral Ecol. 27, 291–310. 10.1046/j.1442-9993.2002.01181.x

[B66] Nevo E. Fu Y. B. Pavlicek T. Khalifa S. Tavasi M. Beiles A. (2012). Evolution of wild cereals during 28 years of global warming in Israel. Proc. Natl. Acad. Sci. U. S. A. 109, 3412–3415. 10.1073/pnas.1121411109 22334646 PMC3295258

[B67] Nicotra A. B. Atkin O. K. Bonser S. P. Davidson A. M. Finnegan E. J. Mathesius U.. (2010). Plant phenotypic plasticity in a changing climate. Trends Plant Sci. 15, 684–692. 10.1016/j.tplants.2010.09.008 20970368

[B68] O'Gorman E. J. Benstead J. P. Cross W. F. Friberg N. Hood J. M. Johnson P. W.. (2014). Climate change and geothermal ecosystems: natural laboratories, sentinel systems, and future refugia. Glob. Change Biol. 20, 3291–3299. 10.1111/gcb.12602 24729541

[B69] Olsen S. L. Topper J. P. Skarpaas O. Vandvik V. Klanderud K. (2016). From facilitation to competition: temperature-driven shift in dominant plant interactions affects population dynamics in seminatural grasslands. Glob. Change Biol. 22, 1915–1926. 10.1111/gcb.13241 26845378

[B70] Orive M. E. Holt R. D. Barfield M. (2019). Evolutionary rescue in a linearly changing environment: limits on predictability. Bullet. Math. Biol. 81, 4821–4839. 10.1007/s11538-018-0504-5 30218277

[B71] Peniston J. H. Barfield M. Gonzalez A. Holt R. D. (2020). Environmental fluctuations can promote evolutionary rescue in high-extinction-risk scenarios. Proc. Royal Soc. B Biol. Sci. 287:9. 10.1098/rspb.2020.1144 32752990 PMC7575515

[B72] Peterson M. L. Angert A. L. Kay K. M. (2020). Experimental migration upward in elevation is associated with strong selection on life history traits. Ecol. Evol. 10, 612–625. 10.1002/ece3.5710 32015830 PMC6988539

[B73] Plue J. Vandepitte K. Honnay O. Cousins S. A. O. (2017). Does the seed bank contribute to the build-up of a genetic extinction debt in the grassland perennial *Campanula rotundifolia*? Ann. Bot. 120, 373–385. 10.1093/aob/mcx057 28645141 PMC5591413

[B74] Pratt J. D. Mooney K. A. (2013). Clinal adaptation and adaptive plasticity in *Artemisia californica*: implications for the response of a foundation species to predicted climate change. Glob. Change Biol. 19, 2454–2466. 10.1111/gcb.12199 23505064

[B75] R Development Core Team (2011). A Language and Environment for Statistical Computing. Vienna: R Foundation for Statistical Computing.

[B76] Ravenscroft C. H. Fridley J. D. Grime J. P. (2014). Intraspecific functional differentiation suggests local adaptation to long-term climate change in a calcareous grassland. J. Ecol. 102, 65–73. 10.1111/1365-2745.12168

[B77] Rinkevich B. (2019). Coral chimerism as an evolutionary rescue mechanism to mitigate global climate change impacts. Glob. Change Biol. 25, 1198–1206. 10.1111/gcb.14576 30680858

[B78] Rolhauser A. G. Nordenstahl M. Aguiar M. R. Pucheta E. (2019). Community-level natural selection modes: a quadratic framework to link multiple functional traits with competitive ability. J. Ecol. 107, 1457–1468. 10.1111/1365-2745.13094

[B79] Saleska S. R. Shaw M. R. Fischer M. L. Dunne J. A. Still C. J. Holman M. L.. (2002). Plant community composition mediates both large transient decline and predicted long-term recovery of soil carbon under climate warming. Glob. Biogeochem. Cycles 16:19. 10.1029/2001GB001573

[B80] Scherrer D. Korner C. (2010). Infra-red thermometry of alpine landscapes challenges climatic warming projections. Glob. Change Biol. 16, 2602–2613. 10.1111/j.1365-2486.2009.02122.x

[B81] Shao H. Xia T. T. Wu D. L. Chen F. J. Mi G. H. (2018). Root growth and root system architecture of field-grown maize in response to high planting density. Plant Soil 430, 395–411. 10.1007/s11104-018-3720-8

[B82] Skalova H. (2010). Potential and constraints for grasses to cope with spatially heterogeneous radiation environments. Plant Ecol. 206, 115–125. 10.1007/s11258-009-9628-x

[B83] Stirbet A Govindjee. (2011). On the relation between the Kautsky effect (chlorophyll a fluorescence induction) and Photosystem II: Basics and applications of the OJIP fluorescence transient. J. Photochem. Photobiol. B. 104, 236–57. 10.1016/j.jphotobiol.2010.12.010 21295993

[B84] Stojanova B. Kolaríková V. Šurinová M. Klápště J. Hadincová V. Münzbergová Z. (2019). Evolutionary potential of a widespread clonal grass under changing climate. J. Evolution. Biol. 32, 1057–1068. 10.1111/jeb.13507 31287927

[B85] Stojanova B. Šurinová M. Klápště J. Koláríková V. Hadincová V. Münzbergová Z. (2018). Adaptive differentiation of *Festuca rubra* along a climate gradient revealed by molecular markers and quantitative traits. PLoS ONE 13:194670. 10.1371/journal.pone.0194670 29617461 PMC5884518

[B86] Šurinová M. Hadincová V. Vandvik V. Münzbergová Z. (2019). Temperature and precipitation, but not geographic distance, explain genetic relatedness among populations in the perennial grass *Festuca rubra*. J. Plant Ecol. 12, 730–741. 10.1093/jpe/rtz010

[B87] Thomann M. Imbert E. Engstrand R. C. Cheptou P. O. (2015). Contemporary evolution of plant reproductive strategies under global change is revealed by stored seeds. J. Evolution. Biol. 28, 766–778. 10.1111/jeb.12603 25682981

[B88] Topper J. P. Meineri E. Olsen S. L. Rydgren K. Skarpaas O. Vandvik V. (2018). The devil is in the detail: nonadditive and context-dependent plant population responses to increasing temperature and precipitation. Glob. Change Biol. 24, 4657–4666. 10.1111/gcb.14336 29851242

[B89] Valladares F. Wright S. J. Lasso E. Kitajima K. Pearcy R. W. (2000). Plastic phenotypic response to light of 16 congeneric shrubs from a Panamanian rainforest. Ecology. 81, 925–1936. 10.1890/0012-9658(2000)0811925:PPRTLO2.0.CO;2

[B90] van Kleunen M. Fischer M. (2005). Constraints on the evolution of adaptive phenotypic plasticity in plants. New Phytol. 166, 49–60. 10.1111/j.1469-8137.2004.01296.x 15760350

[B91] van Kleunen M. Fischer M. (2007). Progress in the detection of costs of phenotypic plasticity in plants. New Phytol. 176, 727–730. 10.1111/j.1469-8137.2007.02296.x 17997755

[B92] Vandvik V. Klanderud K. Meineri E. Maren I. E. Topper J. (2016). Seed banks are biodiversity reservoirs: species-area relationships above versus below ground. Oikos 125, 218–228. 10.1111/oik.02022

[B93] Vandvik V. Klanderud K. Skarpaas O. Telford R. Halbritter A. Goldberg D. (2020). Biotic rescaling reveals importance of species interactions for variation in biodiversity responses to climate change. Proc. Natl. Acad. Sci. U. S. A. 17, 22858–22865. 10.1073/pnas.2003377117 32868426 PMC7502702

[B94] Wilczek A. M. Cooper M. D. Korves T. M. Schmitt J. (2014). Lagging adaptation to warming climate in *Arabidopsis thaliana*. Proc. Natl. Acad. Sci. U. S. A. 111, 7906–7913. 10.1073/pnas.1406314111 24843140 PMC4050579

[B95] Ye X. H. Yu F. H. Dong M. (2006). A trade-off between guerrilla and phalanx growth forms in *Leymus secalinus* under different nutrient supplies. Ann. Bot. 98, 187–191. 10.1093/aob/mcl086 16687430 PMC2803537

