# Supplementary material for: Evolutionary Rescue as a Mechanism Allowing a Clonal Grass to Adapt to Novel Climates
Source: Front Plant Sci. 2021 May 17;12:659479. doi: 10.3389/fpls.2021.659479 (PMC8166245; doi:10.3389/fpls.2021.659479)
Supplement: Supplementary file 1 [file Data_Sheet_1.pdf]

Supporting Information 1. Example of a transplanting order along the transects. The example assumes 2 source populations (S1, S2), 5 clones per source (C1-C5) and two replicates (R1, R2). See methods for the logic of the randomizations.

S1_C3_R1
S2_C5_R1
S2_C2_R1
S1_C4_R1
S1_C4_R1
S2_C1_R1
S1_C1_R1
S2_C2_R1
S2_C3_R1
S1_C5_R1
S1_C2_R2
S2_C4_R2
S2_C5_R2
S1_C5_R2
S2_C1_R2
S1_C4_R2
S1_C1_R2
S2_C3_R2
S1_C3_R2
S2_C2_R2

Supporting Information 2. Variance explained by variables significant in Table 1 out of the variance explained by the total model testing each variable together with Ctemp and Cmois alone and in all interactions. Underlined values are for quadratic functions of the variables and the models contained both the liner and quadratic function of the variable and all interactions.

	Plant trait	Shift in	Plast. R2	Max R2
Height:Ctemp		T		
Height:Cmois	Plant height	M		
Height:Ctemp:Cmois		T x M		
Ramet:Ctemp		T		
Ramet:Cmois	Ramet no.	M		
Ramet:Ctemp:Cmois		T x M		
Extra:Ctemp		T		
Extra:Cmois	Extravag. ramets	M		
Extra:Ctemp:Cmois		T x M	**0.27**	**0.18**
Above:Ctemp		T		
Above:Cmois	Aboveg. biom.	M	**0.25**	
Above:Ctemp:Cmois		T x M		**0.32**
Ratio:Ctemp		T		
Ratio:Cmois	Root:shoot ratio	M		
Ratio:Ctemp:Cmois		T x M		**0.20**
Osmotic:Ctemp		T		
Osmotic:Cmois	Osmotic potential	M	**0.17**	**0.26**
Osmotic:Ctemp:Cmois		T x M	**0.21**	

Supporting Information 3. Effects of quadratic function of plant traits (A) plasticity in osmotic potential, B) maximum of proportion of extravaginal ramets and C) maximum of aboveground biomass measured within previous growth chamber experiments (Münzbergová *et al.* 2017; Kosová *et al.* 2020) on survival of ramets within the field transplant experiment under different levels of moisture and temperature. The values represent mean±SE.

A)

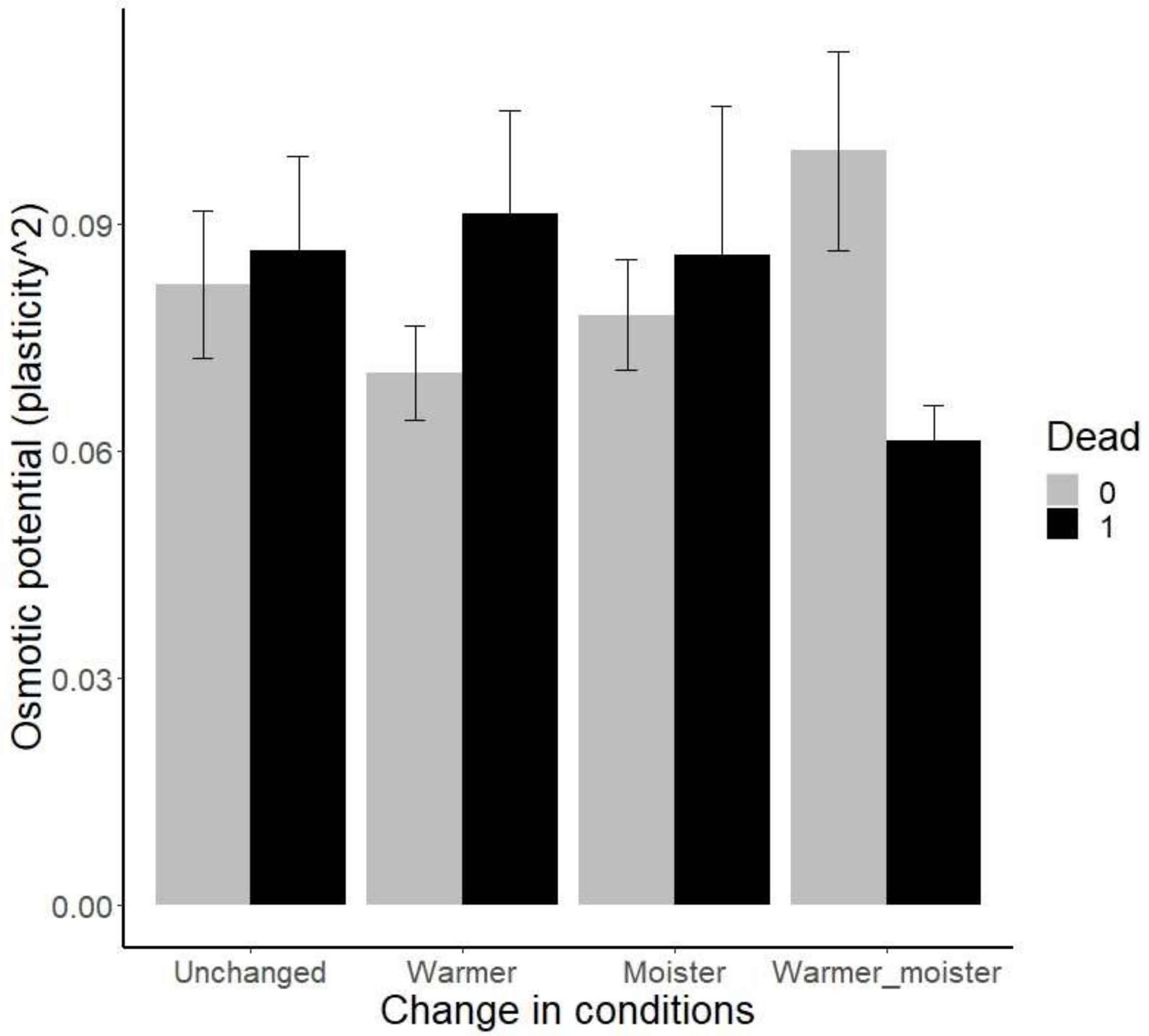

B)

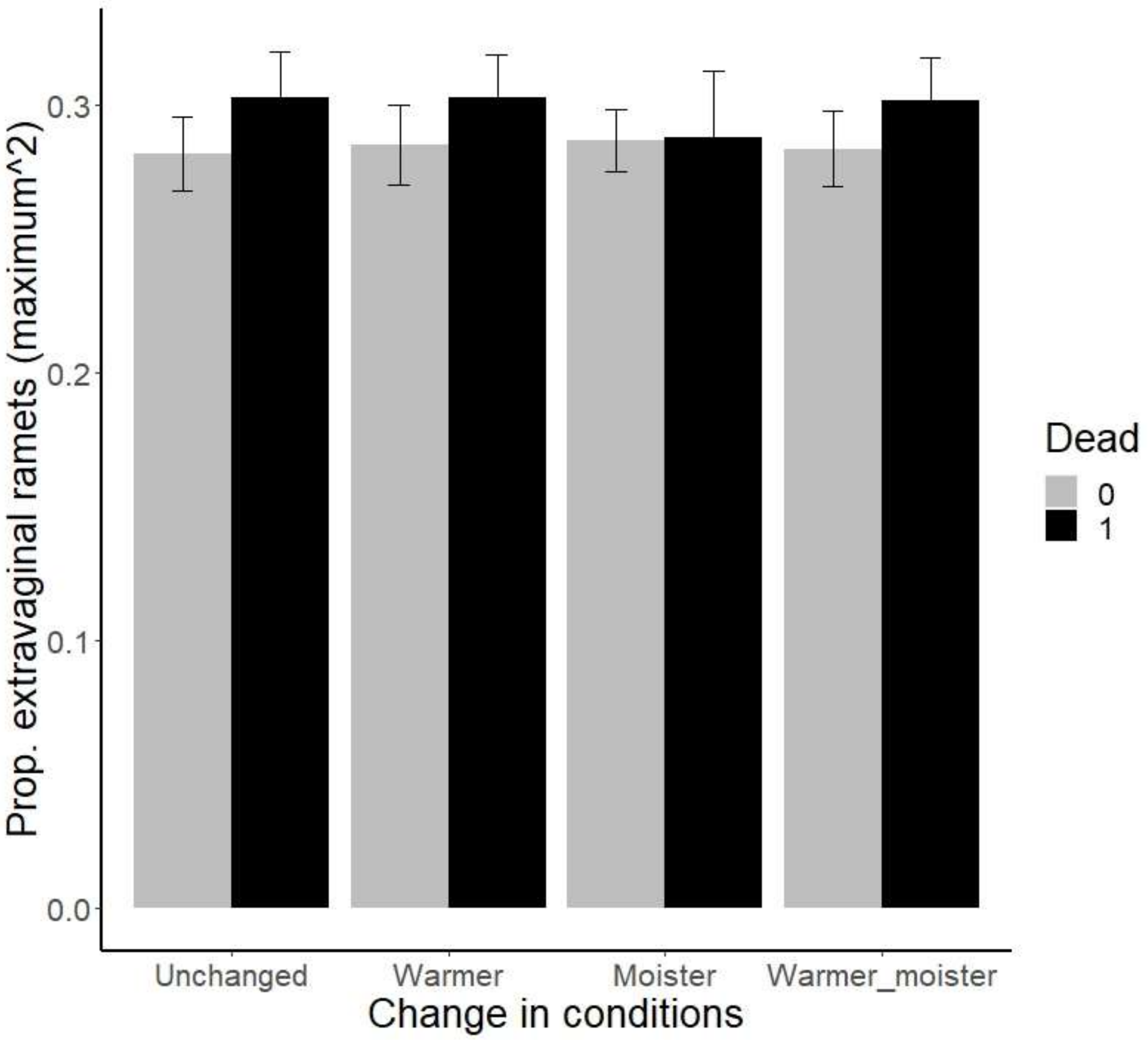
Prop. extravaginal ramets (maximum^2)
0.3
0.2
0.1
0.0
Unchanged
Warmer
Moister
Warmer_moister
Change in conditions
Dead
0
1

c)

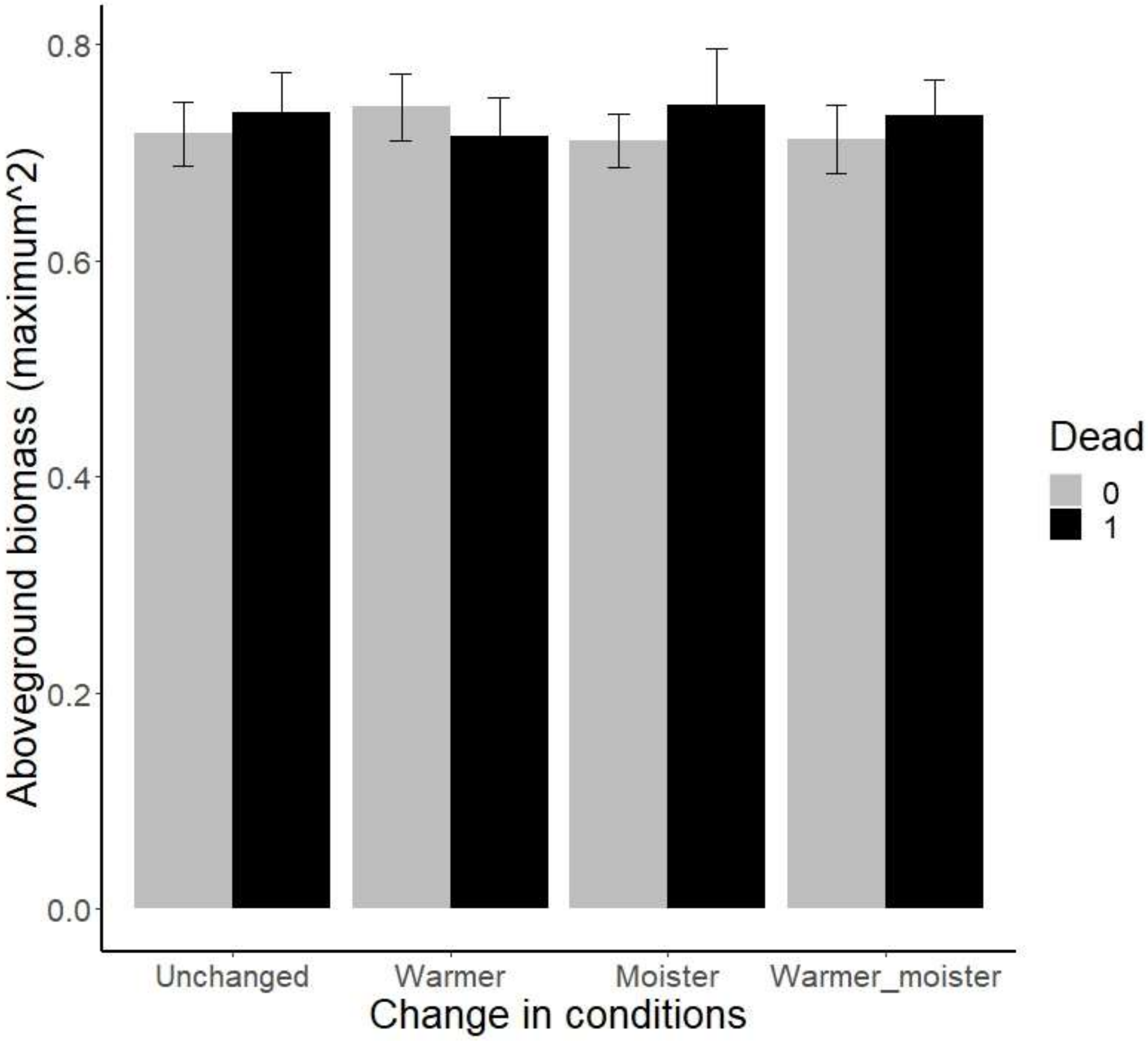
Aboveground biomass (maximum^2)
0.0
0.2
0.4
0.6
0.8
Unchanged
Warmer
Moister
Warmer_moister
Change in conditions
Dead
0
1
